# Early Mortality and Health Care Costs in Patients Recently Diagnosed With Kaposi Sarcoma at the National Cancer Institute, Mexico City

**DOI:** 10.1093/ofid/ofae648

**Published:** 2024-11-09

**Authors:** Daniel Carpio-Guadarrama, Antonio Camiro-Zúñiga, Renzo Pérez-Dorame, Alexandra Martin-Onraët, Diana García-Escutia, María José Mendoza-Palacios, Patricia Volkow-Fernández

**Affiliations:** Infectious Diseases Department, Instituto Nacional de Cancerología, Mexico City, Mexico; Infectious Diseases Department, Instituto Nacional de Cancerología, Mexico City, Mexico; Infectious Diseases Department, Instituto Nacional de Cancerología, Mexico City, Mexico; Infectious Diseases Department, Instituto Nacional de Cancerología, Mexico City, Mexico; Infectious Diseases Department, Instituto Nacional de Cancerología, Mexico City, Mexico; Infectious Diseases Department, Instituto Nacional de Cancerología, Mexico City, Mexico; Infectious Diseases Department, Instituto Nacional de Cancerología, Mexico City, Mexico

**Keywords:** DALYs, health care cost, HIV mortality, Kaposi sarcoma, YLL

## Abstract

**Background:**

Kaposi sarcoma (KS) is a marker of advanced HIV disease; it is still the most frequent AIDS-associated malignancy in Mexico despite universal access to antiretroviral therapy, reflecting a gap in early HIV diagnosis.

**Methods:**

The objectives of the study were to describe people with HIV with KS who died within 30 days of admission at INCan (National Cancer Institute) and to quantify resources and years of life lost (YLL). We collected demographic data, HIV-related variables, all diagnostic and therapeutic procedures, hospitalizations, and estimated YLL and disability-adjusted life years.

**Results:**

Eighteen (6.7%) people with HIV with KS from 270 patients admitted at INCan from 2014 to 2021 were included. The median age was 31 years (IQR 27–36), and the median days from admission to death and from HIV diagnosis to death were 15 (IQR, 6–24) and 73 (IQR, 30–857), respectively. Upon admission, the median HIV viral load was 314 476 copies/mL (IQR, 140 709–695 613); CD4+ T cells, 93 cells/mL (IQR 35–124); and CD4/CD8 ratio, 0.08 (IQR, 0.06–0.12). Coinfections were diagnosed in 14 (77.7%) patients. The average expenditure per patient was US $7685.99 USD, and the total YLL was 737.4 with a median 42 years (IQR, 37.7–47) per patient. The total care cost was US $183 947.48, equivalent to a screening program in key populations, which would have allowed the early detection of 1227 cases and saved 8410 disability-adjusted life years.

**Conclusions:**

Reinforcement of early HIV infection detection in key population programs should be prioritized to reduce KS-associated deaths and YLL and for rational use of health budgets.

Kaposi sarcoma (KS) was 1 of the 2 markers that heralded the beginning of the HIV pandemic in 1981 [1, 2]. The incidence was 10 000 times higher in people with HIV (PWH) as compared with the general population [3], and the presentation of the disease was usually very aggressive, with high early mortality [4]. Early antiretroviral therapy (ART) and timely HIV diagnoses radically reduce KS incidence and mortality. As a result, KS incidence has dramatically dropped in most high-income countries with access to universal ART and robust HIV detection programs, and it is no longer the most frequent AIDS-related malignancy [5]. For example, in the United States, HIV-related KS incidence has shown a reduction of 70%, and Swiss cohorts have seen almost an 8-fold drop in the standardized incidence ratio for KS [5, 6].

In Latin America, although most countries guarantee universal access to ART, HIV detection programs have lagged. Consequently, late presentation to HIV care services is still highly prevalent [7]. As a result, patients with KS often present to care with advanced stages of the disease and experience a high burden of morbidity and mortality [7, 8]. For example, in Mexico's largest referral national center for PWH and cancer, KS remains the most frequent malignancy in PWH even after 20 years of universal ART access in the country [8].

For these reasons, this study aims to describe the clinical characteristics of PWH referred to our institution with KS who died within 30 days of hospital admission and to estimate the extent of resources spent on their care during their hospital stay, as well as to calculate years of life lost and the disability-adjusted life years (DALYs) that could have been averted if a similar number of resources was spent in early HIV detection programs.

## METHODS

We conducted a retrospective cohort study that included all PWH with KS treated at the National Cancer Institute (INCan) in Mexico City from 2014 to 2021. The INCan is a national health institute that is part of the Mexican Ministry of Health and has provided care to people without social security utterly free of charge since 2018.

The Institutional Research Committee of the Instituto Nacional de Cancerología (No. 2024/020; Comité de Ética en Investigación, México) approved the study and waived informed consent. This retrospective study consists of information obtained from clinical charts; patient confidentiality was maintained.

To carry out the study, we identified all individuals in the cohort who died within 30 days of arrival for care at our institution. Patients who have abandoned ART returning to care, or naive were included. We collected sociodemographic data, substance abuse history, data related to HIV disease status and treatment, data regarding diagnostic procedures for opportunistic infections and their treatment as well as other sexually transmitted diseases, and data regarding routine in-hospital care and hospitalization length. We calculated all expenses generated by each patient during hospitalization using the recorded variables and standard cost tables for services and medications used by the National Health Ministry to calculate the bill of noninsured patients. If standard costs for a particular service or supply had not been updated for 2024, we adjusted for the compound national inflation index since the last year that said value was updated. In the particular case of blood derivates for transfusions—which, by Mexican law, are provided free and through altruistic donations since the 1987 and thus have no calculated value—we used a cost calculation study that estimates the expenditure required for this service in the public health ministry [9] and adjusted it for the compound national inflation index since the year that it was published.

We calculated years of life lost by subtracting the patient's age from the life expectancy estimate given to Mexico by the World Bank for the year when the patient died [10]. We estimated DALYs that could have been gained if a similar amount of resources had been spent early on HIV detection programs by extrapolating the total expenditures used for the attention of the included population and converting them to their equivalent cost in counseling sessions for key populations. Expenditures were converted from Mexican pesos to US dollars by the exchange rate of the date of the main analysis. The standard cost in US dollars for a counseling session was taken as the average cost for a counseling session in the meta-analysis by Pineda-Antunez et al [11]. The possible early HIV diagnoses that could be obtained with these counseling sessions were calculated by assuming that they were undertaken in key populations, with a high HIV incidence, and that this population would not be reached in a timely manner otherwise. We assumed a median age of HIV diagnosis of 35 years and an HIV prevalence of 11.9% in key populations, according to the 2022 UNAIDS estimates for our country [12]. The total possible DALYs averted were calculated by subtracting the DALYs generated by a pre-AIDS diagnosis in the calculated possible-to-reach population (0.078 DALYs by year of life) from the DALYs generated by an AIDS case that receives ART in the same population (0.274 DALYs by year of life), representing the difference in DALYs from late presentation to early detection [13]. To calculate the years of life for the DALY generation, we assumed a median age of HIV diagnosis of 35 years (according to the 2022 UNAIDS estimates for our country) [12] and a life expectancy of 70 years [10]. We did a second DALY calculation assuming a 20% loss to follow-up to provide a range of possible scenarios that could better reflect the real-world impact of early HIV detection in our population.

## RESULTS

During the study period, 270 patients were referred to INCan with a diagnosis of disseminated KS, and 18 patients met the inclusion criteria, representing 6.7%. All were male. The median age at admission was 31 years (IQR, 27–36); 88.8.4% identified as men who have sex with men (MSM). Upon admission, patients had a median weight of 55 kg (IQR, 50–62.3) and a body mass index of 20 (IQR, 18.4–22). Ten (55.5%) had a history of consuming some substance, with tobacco being the most prevalent (55.5%), followed by alcohol (44.4%), marijuana (16.6%), and cocaine (22.2%).

Upon admission, the median HIV viral load was 314 476 copies/mL (IQR, 140 709–695 613); the CD4+ T-cell count was 93 cells/mL (IQR, 35–124); and the CD4/CD8 ratio was 0.08 (IQR, 0.06–0.12). Six (33.3%) patients reported a history of ART abandonment with a median suspension duration of 21 months (IQR, 18–36). There were no statistical differences in the clinical variables of patients who were ART experienced or naive to ART. Thirteen (72.2%) patients got to initiate ART, with 9 (50%) of them doing so before admission to our institution and 4 (22.2%) during their hospitalization. The median time from antiretroviral initiation to death was 44 days (IQR, 15–104). Three (16.6%) patients were receiving steroid therapy on arrival: 1 died 6 days and 2 died 7 days after the date of arrival.

**Figure 1.**
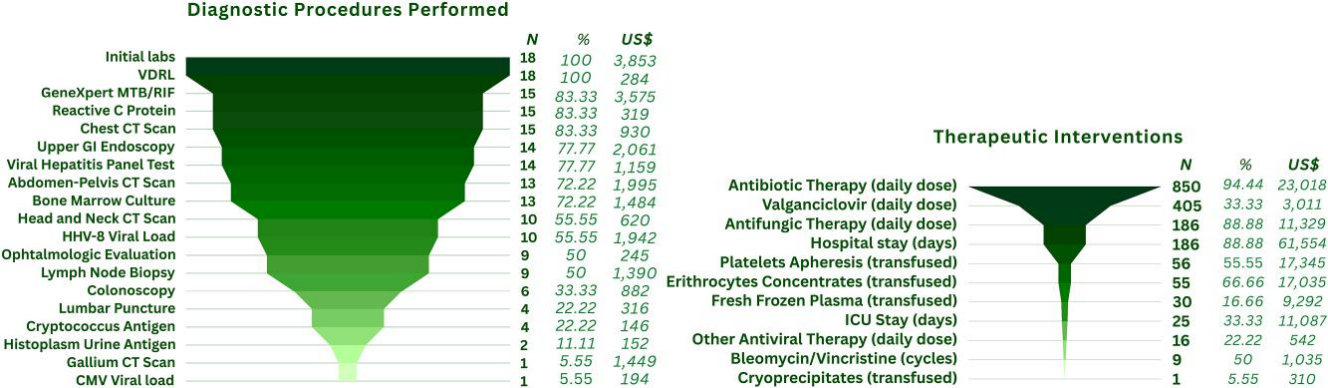
The diagnostic and therapeutic procedures, as well as the hospital resources used, in the care of patients with disseminated Kaposi sarcoma who died within 30 days of arrival at the Instituto Nacional de Cancerología. CMV, cytomegalovirus; CT, computed tomography; GI, gastrointestinal; HHV-8, human herpesvirus 8; ICU, intensive care unit; VDRL, venereal disease research laboratory.

Figure 1 depicts diagnostic procedures performed on the entire group, including imaging studies and searches for coinfections. Coinfections were diagnosed in 14 (78%) patients with the following distribution: syphilis (27.7%), candidiasis (27.7%), hepatitis B (16.6%), cytomegalovirus end-organ disease (22.2%), histoplasmosis (11.1%), cryptococcosis (5.5%), and 1 patient with extended-spectrum β-lactamase *Klebsiella pneumoniae* and *Penicillium* pleural infection. Treatment was initiated according to each pathology. There was 1 case of *Mycobacterium avium* complex (7.7%) in 13 bone marrow cultures; the result was obtained after the patient's death. Nine (50%) patients received 1 chemotherapy cycle with bleomycin/vincristine. Two patients had received previous regimens before admission with liposomal-doxorubicin at 1 and 5 cycles. Eleven patients received blood component transfusions from 56 platelet apheresis units, 55 red blood cell packages, 30 fresh-frozen plasmas, and 1 cryoprecipitate.

The median days from admission to death and from HIV diagnosis to death were 15 (IQR, 6–24) and 73 (IQR, 30–857), respectively. The sum of the years of life lost in our patient population is equivalent to 737.4 years [12], with a median of 42 (IQR, 37.7–47) per patient.

**Figure 2.**
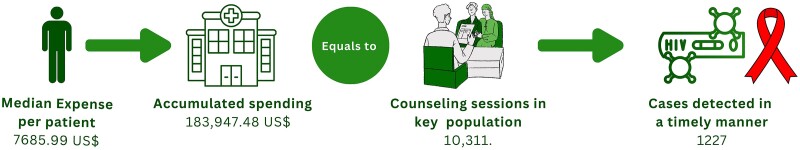
The equivalence of health expenses in 2 scenarios: treatment of advanced HIV–Kaposi sarcoma disease vs consulting session for HIV detection in key populations.

In the resource consumption analysis, the average expenditure per patient was US $7685.99 (exchange rate for 30 May 2024, 16.95 pesos per US dollar), with a total spending of US $183 947.48 for the 18 patients, adjusted for inflation (Figure 1). The rate of expenditure per hospitalization day was US $988.58. The calculated cost of a counseling session with an HIV screening test in key populations such as MSM, sex workers, people who inject drugs, people in prisons, and transgender people [11, 14] was estimated at US $17.84 per session, which includes a third-generation HIV rapid test and a session with a counselor [11]. The total sum of expenses, when converted to its equivalent in HIV counseling sessions, could allow for the implementation of 10 311 counseling sessions and the possible timely detection of an estimated 1227 new HIV cases, at 68 cases per care expenditure for a PWH with disseminated KS (Figure 2). The expected spared DALYs for these early detections would be 8410.4: 11 757.3 DALYs averted by prevented AIDS cases minus 3345 DALYs generated by the equivalent population with pre-AIDS diagnosis. This represents an expenditure of US $21.86 per averted DALY, which increases to US $27.32, assuming a 20% loss to follow-up.

## DISCUSSION

In this study, we describe the costs associated with the hospitalization and care of individuals with disseminated KS who died during their initial presentation to care at INCan in Mexico City. We calculated an average expenditure per patient of roughly US $10 000. The investment of the same amount in HIV counseling sessions would allow for the timely detection of 68 new cases of HIV in key populations per disseminated KS patient expenditure for an estimated total of 1227 early diagnoses and 8410 spared DALYs.

Almost 7% of patients presenting with KS died within the first 30 days of arrival at our institute. Our findings are similar to the reported survival curves of other cohorts in individuals with KS in Africa and Latin America. In a retrospective study in South Africa, Chu et al [13] describe 215 individuals with HIV-associated KS from May 2001 to January 2007. The median age and CD4 count at presentation were similar to those in our study (34 years and 82 cells/mL). Still, the authors did not report coinfections or clinical status, other than KS staging. A prospective study in South Africa [15] describes a cohort of 13 847 PWH. Age and CD4 count at presentation were similar to our study (35 years and 74 cells/mL), but tuberculosis coinfection was more frequent (37% vs 5.2%). Both studies found a mortality rate of around 5% to 10% at the 1-month mark. Finally, in a multicentric study by Castilho et al [15] analyzing 481 individuals with KS in several Latin American centers (including 51 Mexican patients), the mortality rate was roughly 5% at the 1-month mark, with similar ages and CD4 counts. Similar findings in age, CD4 count, and mortality had been informed by our group [16].

Although it is difficult to individually assess the direct impact of social and economic factors on mortality in the studied population, we can use some surrogate indicators of social and economic vulnerability [17, 18]. All patients included in the study were part of key populations (MSM and young people) and had multiple sexually transmitted diseases (especially syphilis), and around half presented with a history of various substance abuse. Most important of all, all deceased individuals included in the study were late presenters and had advanced HIV disease, which has been described as the most important determinant in survival in this population in our region and is strongly correlated to vulnerability [19]. This has substantial implications: it suggests that interventions directed to HIV prevention, in previously described vulnerable populations in our country, present an excellent opportunity to avert disseminated KS and reduce the attention costs and mortality in individuals with similar socioeconomic backgrounds.

Situations that increase mortality in individuals with KS are related to the severe state of immunodeficiency, as was the case in this cohort in whom the CD4/CD8 ratio was <0.1; this severe immunodeficiency environment is essential for human herpesvirus 8 to produce KS.

Access to ART has reduced KS incidence in PWH; however, in patients with disseminated KS, mortality increases in the first weeks after initiation of ART [14, 15], as related to immune reconstitution inflammatory syndrome, which has been associated with early high mortality [17]. In our cohort, 73.7% of patients with KS had initiated ART, with 8 starting before arrival at our institution, with a median 58 days (IQR, 26–158).

Treatment with glucocorticoids has been associated with worse clinical outcomes due to increased KS cell proliferation, as it increases spindle cell proliferation signaling [19, 20]; 3 patients in our cohort had received steroids and all died in the first week of arrival. Finally, severe opportunistic infections were detected in 3 patients: 2 with disseminated histoplasmosis and 1 with meningeal cryptococcosis. Still, specific therapy was promptly initiated, except for a patient with *M avium* complex, whose diagnosis was established 7 days after his death. All of this suggests that mortality was mainly driven by the advanced stage of KS per se and infectious comorbidities.

Immune reconstitution inflammatory syndrome–KS and other AIDS-defining events in patients with KS are associated with early mortality. This is why we have established a thorough clinical evaluation on arrival at our institution, including human herpesvirus 8 viral load and extensive workup to diagnose and treat coinfections and rule out other neoplasms [21]. This protocol is started on patient arrival for care and has been proven to diminish immune reconstitution inflammatory syndrome–KS and mortality. In this context, the consumption of diagnostic, therapeutic, and hospital supplies associated with KS care was substantial, with a median US $7686 per patient. To put these numbers in perspective, we compare our KS health care costs model with the costs of cervical cancer care (a preventable disease). Using surrogate data from other countries due to a lack of local data, we can roughly estimate that the associated inpatient expenses in cervical cancer are similar to the per-patient expense for KS found in our study (median, US $12 550 from models based on MarketScan databases from 2008 to 2016 in the United States vs US $7686 for KS in our study) [22]. The costs associated with cervical cancer prevention (Papanicolaou smear and histology examination) are higher (median, US $60.14 vs $17.84 as used in HIV counseling sessions based on the French public health care system models) [23]. The proposed models for the cost-effectiveness of generalized Papanicolaou smear interventions for cervical cancer prevention calculate an inflation-corrected rate of US $1433.93 (exchange rate for 30 May 2024, 16.95 Mexican pesos per US dollar) per averted DALY [24] (vs US $22–27.6 per averted DALY in our KS model). This shows that improving funding in early HIV detection programs is cost-effective and comparable to other similar prevention programs in at-risk populations. At the time of writing, we did not find any other studies reporting on the associated health care costs of HIV-related KS, so we could not make comparisons with other health care systems or populations. However, it has consistently been recognized that the most essential tools to reduce KS incidence are HIV diagnosis and ART initiation and maintaining high CD4 cell counts [25].

This study has some significant limitations. It is a small sample from a single reference center and might not adequately represent the whole country or other regions. A bigger sample would have probably provided more accurate data to improve DALY calculations. As our institution is a tertiary care hospital specialized in HIV and malignancies, we receive patients with advanced disease who are mostly referred from primary HIV care centers: Clínica Especializada Condesa, Mexico City and CAPASITS in other states of the country.

Diagnostic and therapeutic procedures were not standardized until 2020; as such, in the earlier period, the treating medical team's criteria and possible biases heavily dictated the resources spent in each case, with an important amount of subsequent variation among individuals. We did not consider other possible important variables with an impact on mortality, as all patients had disseminated KS and presented with severe wasting and deteriorated nutritional status. Also, we did not consider other interventions’ costs in our model of early detection that could elevate the total expenses of detecting patients in a pre-AIDS stage, such as ART and the cost of linking to care. Finally, autopsies were not undertaken on any patient, so the cause of death remains based only on clinical and laboratory test results.

## CONCLUSION

Our findings emphasize the urgent need to reinforce programs in key populations for early HIV infection diagnosis and timely combination ART initiation. Such measures could significantly reduce mortality, improve quality of life, and lower health care costs in patients who are HIV positive. Barriers to early diagnosis and treatment, such as stigma and obstacles to reaching health care access, should be prioritized to improve outcomes in this vulnerable population.

## Notes

***Financial support.*** No funding was received for this study.
